# Exploring the integration of medical and preventive chronic disease health management in the context of big data

**DOI:** 10.3389/fpubh.2025.1547392

**Published:** 2025-04-15

**Authors:** Yueyang Wang, Ruigang Deng, Xinyu Geng

**Affiliations:** ^1^Office of Medical Defense Integration, The Fourth People's Hospital of Sichuan Province, Chengdu, China; ^2^School of Computer Science and Software Engineering, Southwest Petroleum University, Chengdu, China

**Keywords:** chronic disease management, health care and prevention integration, risk prediction modeling, big data, preventive management

## Abstract

Chronic non-communicable diseases (NCDs) pose a significant global health burden, exacerbated by aging populations and fragmented healthcare systems. This study employs a comprehensive literature review method to systematically evaluate the integration of medical and preventive services for chronic disease management in the context of big data, focusing on pre—hospital risk prediction, in—hospital clinical prevention, and post—hospital follow—up optimization. Through synthesizing existing research, we propose a novel framework that includes the development of machine learning models and interoperable health information platforms for real—time data sharing. The analysis reveals significant regional disparities in implementation efficacy, with developed eastern regions demonstrating advanced closed—loop management via unified platforms, while western rural areas struggle with manual workflows and data fragmentation. The integration of explainable AI (XAI) and blockchain—secured care pathways enhances clinical decision—making while ensuring GDPR—compliant data governance. The study advocates for phased implementation strategies prioritizing data standardization, federated learning architectures, and community—based health literacy programs to bridge existing disparities. Results show a 30–35% reduction in redundant diagnostics and a 15–20% risk mitigation for cardiometabolic disorders through precision interventions, providing a scalable roadmap for resilient public health systems aligned with the “Healthy China” initiative.

## 1 Introduction

Chronic non-communicable (NCDs) diseases have become a major disease burden worldwide, imposing tremendous pressure on society and the economy. Meanwhile, the rapid development of information technology has provided new approaches and methods for chronic disease management. This paper reviews the research on the integration of information technology with chronic disease management.

### 1.1 Current status and challenges of chronic diseases

NCDs refer to a range of diseases including diabetes, cardiovascular diseases, chronic respiratory diseases, and malignant tumors. These diseases are the primary health threats to the population, characterized by their insidious onset, complex causes, and prolonged conditions. They involve multiple systems such as the endocrine, cardiovascular, and respiratory systems, with hypertension, hyperglycemia, hyperlipidemia, and hypoglycemia being the most typical manifestations. According to the World Health Organization’s (WHO) “World Health Statistics 2023”report (1), NCDs have caused the highest disease burden globally. In 2000, 61% of deaths worldwide (31 million) were attributed to NCDs, and this proportion increased to 74% (41 million) by 2019. Additionally, the “Research on the Development Prospects of the Chronic Disease Management Industry in China” (2) by the China Industry Research Network points out that China has fully entered an aging society. It is estimated that by 2025, the older adult population in China will exceed 200 million, by 2035 it will exceed 300 million, and by 2050 it will reach 380 million. In China, the proportion of deaths caused by chronic diseases is as high as 86%, with an increasingly severe disease burden. To improve chronic disease management in China, the General Office of the State Council issued the “Mid- and Long-Term Plan for the Prevention and Treatment of Chronic Diseases in China (2017–2025)” based on the “Healthy China” strategy, focusing on advancing chronic disease management. Therefore, the prevention and control of chronic diseases is a crucial development goal of the 21st century.

### 1.2 The introduction of “integration of medical treatment and prevention” has optimized the chronic disease management model

In 2018, the National Health Commission of China set new requirements for primary healthcare services, introducing “integration of medical treatment and prevention” as a new concept for chronic disease prevention and control. In 2019, the National Health Commission further emphasized in the “Notice on Basic Public Health Service Projects in 2019” that chronic disease management, such as hypertension and diabetes, should be used as an entry point to explore a grassroots service model characterized by “integration of medical treatment and prevention.” In 2023, the “Opinions on Further Deepening Reform and Promoting Healthy Development of Rural Healthcare Systems,” issued by the General Office of the CPC Central Committee and the General Office of the State Council, proposed innovative mechanisms for medical-prevention coordination and integration. It can be said that “integration of medical treatment and prevention” is a relatively new policy concept (3), frequently appearing in policy documents in recent years and garnering significant attention. “Medical” primarily refers to clinical diagnosis and treatment, while “prevention” mainly pertains to public health. Compared to the previous health guideline of prevention-first and combining prevention with treatment, this new approach emphasizes how medical treatment and prevention can be integrated to simultaneously improve technical levels and form a unified management model. The integration of medical treatment and prevention may provide new pathways for reform and innovation in chronic disease management.

### 1.3 Big data-driven chronic disease management

#### 1.3.1 Definition of healthcare big data

**Figure 1:**
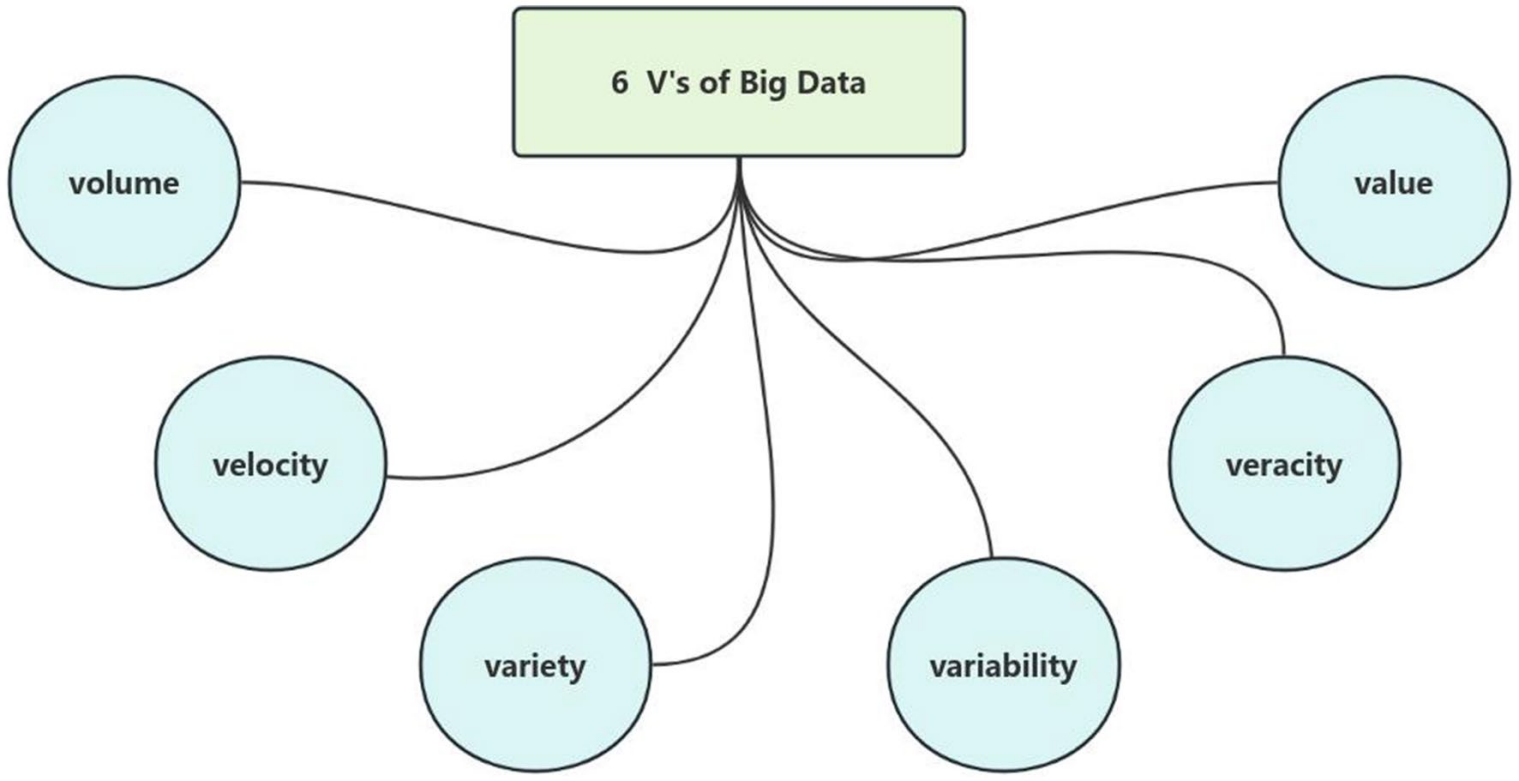
6 V’s of big data.

Big data refers to datasets characterized by massive volume, heterogeneous variety, rapid velocity, and significant variability, which exceed the processing capabilities of traditional database management tools. In the healthcare domain, medical big data similarly exhibits these attributes along with two additional critical dimensions: veracity (data quality) and value (actionable insights), collectively constituting the six defining characteristics of big data (Volume, Velocity, Variety, Variability, Veracity, and Value) (4), as illustrated in Figure 1.

Healthcare big data (5) originates from diverse sources, including but not limited to:

- Electronic Medical Records (EMRs).
- Clinical diagnostic documentation.
- Laboratory and imaging examination reports.
- Pharmaceutical databases.
- Medical device monitoring systems.
- Patient-generated health data via wearable devices.
- Public health surveillance platforms.

These multimodal datasets encompass comprehensive patient health information and clinical workflows, spanning structured formats (e.g., numerical values in EMRs), semi-structured metadata (e.g., DICOM headers in medical imaging), and unstructured content (e.g., physician narratives, diagnostic reports, and radiological images). The inherent heterogeneity and scale of these data types pose significant challenges in data processing and analytical methodologies.

Notably, while healthcare big data demonstrates immense potential for clinical insights, its intrinsic value is critically dependent on data quality (6). The accuracy and reliability of clinical decision-making are fundamentally dependent on rigorous data preprocessing and advanced analytical frameworks. Consequently, systematic data curation and robust computational strategies constitute essential prerequisites for transforming raw healthcare data into clinically actionable knowledge.

#### 1.3.2 Methodological framework for chronic disease modeling using big data

**Figure 2:**
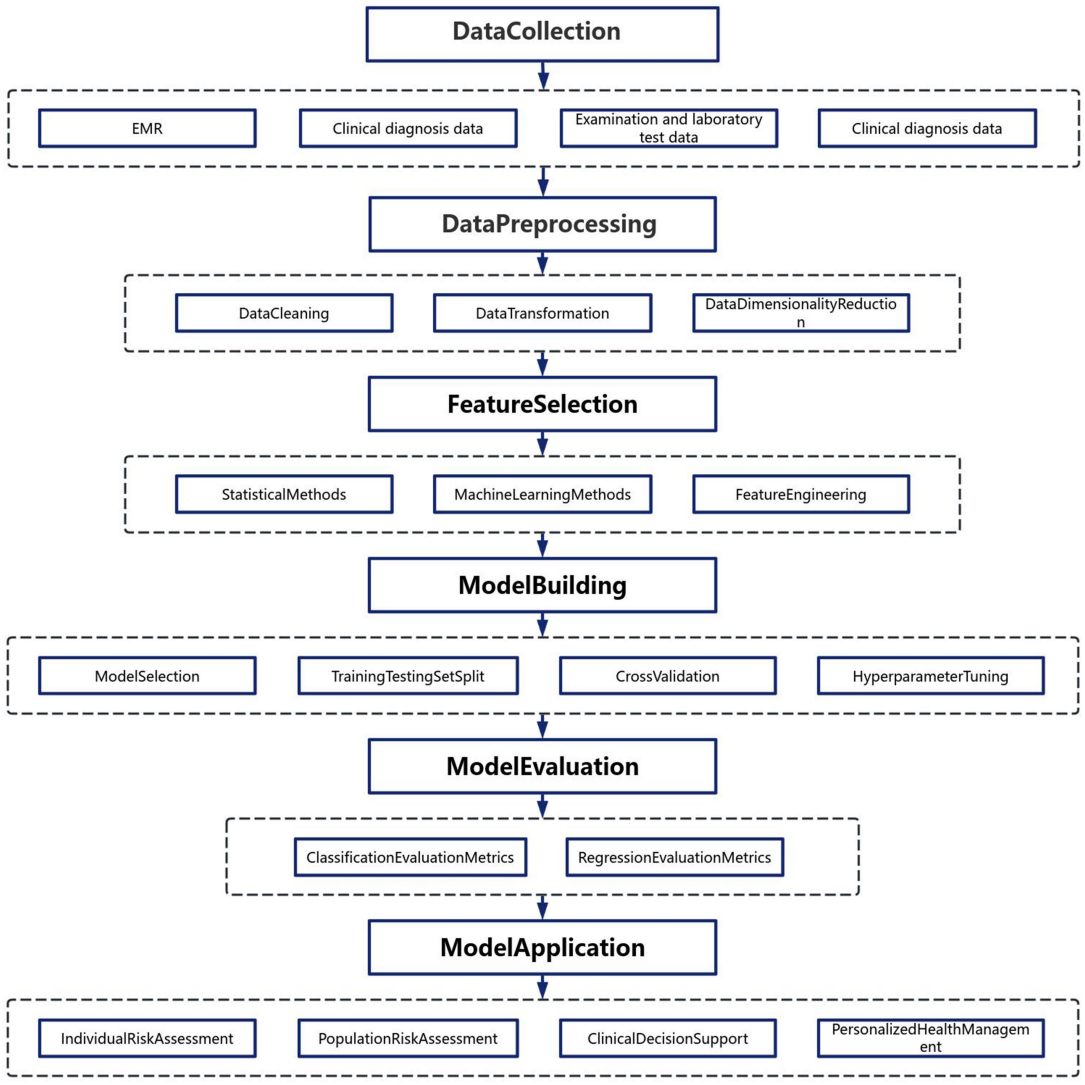
Big Data modeling roadmap.

The rapid advancement of information communication technologies and artificial intelligence has enabled sophisticated methodologies for data processing and analytical modeling. As illustrated in Figure 2, the construction of chronic disease prediction models follows a systematic workflow comprising five critical phases (7):

##### 1.3.2.1 Phase 1: multisource data acquisition

Heterogeneous medical datasets are aggregated from electronic health records, wearable sensors, genomic repositories, and population health databases. These multimodal inputs provide a comprehensive digital representation of patient health trajectories, encompassing demographic, biochemical, imaging, and behavioral dimensions.

##### 1.3.2.2 Phase 2: preprocessing pipeline

Raw data undergoes rigorous preprocessing to ensure analytical validity:

- Data Cleansing: Removal of outliers, imputation of missing values, and correction of erroneous entries.
- Dimensionality Reduction: Application of principal component analysis (PCA) or t-distributed stochastic neighbor embedding (t-SNE) to mitigate the curse of dimensionality.
- Normalization: Standardization/z-score transformation to enhance feature comparability across disparate scales.

##### 1.3.2.3 Phase 3: feature engineering and selection

A dual-strategy approach optimizes predictive features:

1. Statistical Filtering: Chi-square tests and mutual information metrics identify variables with significant associations to chronic disease endpoints.
2. Algorithmic Selection: Recursive feature elimination (RFE) coupled with tree-based classifiers (e.g., XGBoost, LightGBM) quantifies feature importance.
3. Feature augmentation: nonlinear transformations and interaction term generation enhance model expressivity

##### 1.3.2.4 Phase 4: predictive model development

Ensemble learning architectures demonstrate superior performance in handling healthcare data complexity:

- Baseline Models: Logistic regression with L1/L2 regularization establishes performance benchmarks.
- Advanced Architectures: Deep neural networks with attention mechanisms capture temporal dependencies in longitudinal data.
- Validation Protocol: Stratified k-fold cross-validation (k = 5/10) prevents overfitting while maintaining class distribution integrity.
- Hyperparameter Optimization: Bayesian optimization frameworks efficiently navigate high-dimensional parameter spaces.

##### 1.3.2.5 Phase 5: clinical implementation

Deployed models serve multiple translational functions:

- Risk Stratification: Generation of individualized risk scores using SHAP (SHapley Additive exPlanations) values.
- Precision Prevention: Dynamic recommendation systems for lifestyle modifications and therapeutic interventions.
- Population Analytics: Geospatial clustering identifies high-risk cohorts for targeted public health initiatives.

##### 1.3.2.6 Performance evaluation metrics

Model efficacy is quantified through:

- Discrimination: AUC-ROC curves with 95% confidence intervals.
- Calibration: Brier scores and calibration belt analysis.
- Clinical utility: Decision curve analysis quantifying net benefit across risk thresholds.

This methodological framework emphasizes reproducibility through adherence to TRIPOD (Transparent Reporting of a multivariable prediction model for Individual Prognosis or Diagnosis) guidelines, ensuring clinical relevance while maintaining computational rigor (8). The integration of explainable AI (XAI) techniques facilitates clinician trust and regulatory compliance in real-world deployment scenarios.

#### 1.3.3 Potential biases and limitations in integrated clinical-public health data systems

The integration of clinical and public health data systems, while offering significant potential for comprehensive disease surveillance and management, is inherently constrained by systemic biases and methodological limitations. Key challenges arise from heterogeneous data quality across multi-source inputs, including inconsistencies in completeness (e.g., variable documentation practices), accuracy (e.g., divergent diagnostic coding standards), and temporal resolution (e.g., mismatched data collection frequencies between real-time clinical monitoring and periodic public health reporting). These issues are compounded by technical interoperability barriers stemming from incompatible data standards (HL7 FHIR vs. OpenEHR), organizational fragmentation in data governance, and semantic discrepancies between preventive health terminologies and clinical ontologies (9). Furthermore, inherent selection biases may skew analyses, particularly through underrepresentation of marginalized populations in digital health records and confounding effects of differential healthcare-seeking behaviors. To address these challenges, a robust quality assurance framework spanning pre-analytical, analytical, and post-analytical phases is essential. This includes implementing constrained data entry interfaces with real-time validation protocols during data acquisition, deploying machine learning-powered anomaly detection systems for continuous quality monitoring, and conducting root-cause analyses using Bayesian networks to identify systemic errors in data pipelines. Interoperability enhancement requires dual architectural and policy interventions, such as adopting federated learning systems for privacy-preserving distributed analytics, integrating SMART-on-FHIR (10) APIs to bridge clinical and population health platforms, and incentivizing standardized common data models (OMOP CDM, PCORnet) (11). Crucially, these technical solutions must be coupled with rigorous validation metrics—including composite data utility indices assessing completeness (≥95%), accuracy (≥0.85), and timeliness (latency <24 h)—and compliance with international healthcare IT benchmarks (HIMSS EMRAM Stage 6+). The implementation of cryptographic security mechanisms, such as homomorphic encryption for cross-institutional data harmonization, ensures adherence to GDPR and HIPAA regulations while maintaining data utility. This multifaceted approach underscores the necessity of continuous quality improvement cycles and coordinated policy-technical strategies to transform fragmented data ecosystems into reliable, actionable intelligence for integrated care delivery.

In terms of data security, which is equally important, technologies such as blockchain can be employed to ensure data security and compliance. For instance, in cross-border data circulation, federated learning (FL) can be used to train models on decentralized data, ensuring that raw data does not cross borders and thus complying with China’s Cybersecurity Law. To adhere to the General Data Protection Regulation (GDPR), privacy protection techniques such as differential privacy can be adopted during the FL aggregation process, further enhancing data security.

Additionally, the application of auditable compliance blockchain can record data access and processing activities, ensuring the traceability of data usage. For example, the SMART-on-FHIR API (12) can trigger GDPR “right to be forgotten” requests through blockchain entries, safeguarding the legitimate rights and interests of data subjects. Furthermore, the establishment of regional data governance committees, such as the China-EU Health Data Working Group, can help unify data standards and promote data circulation and cooperation between different regions.

**Figure 3:**
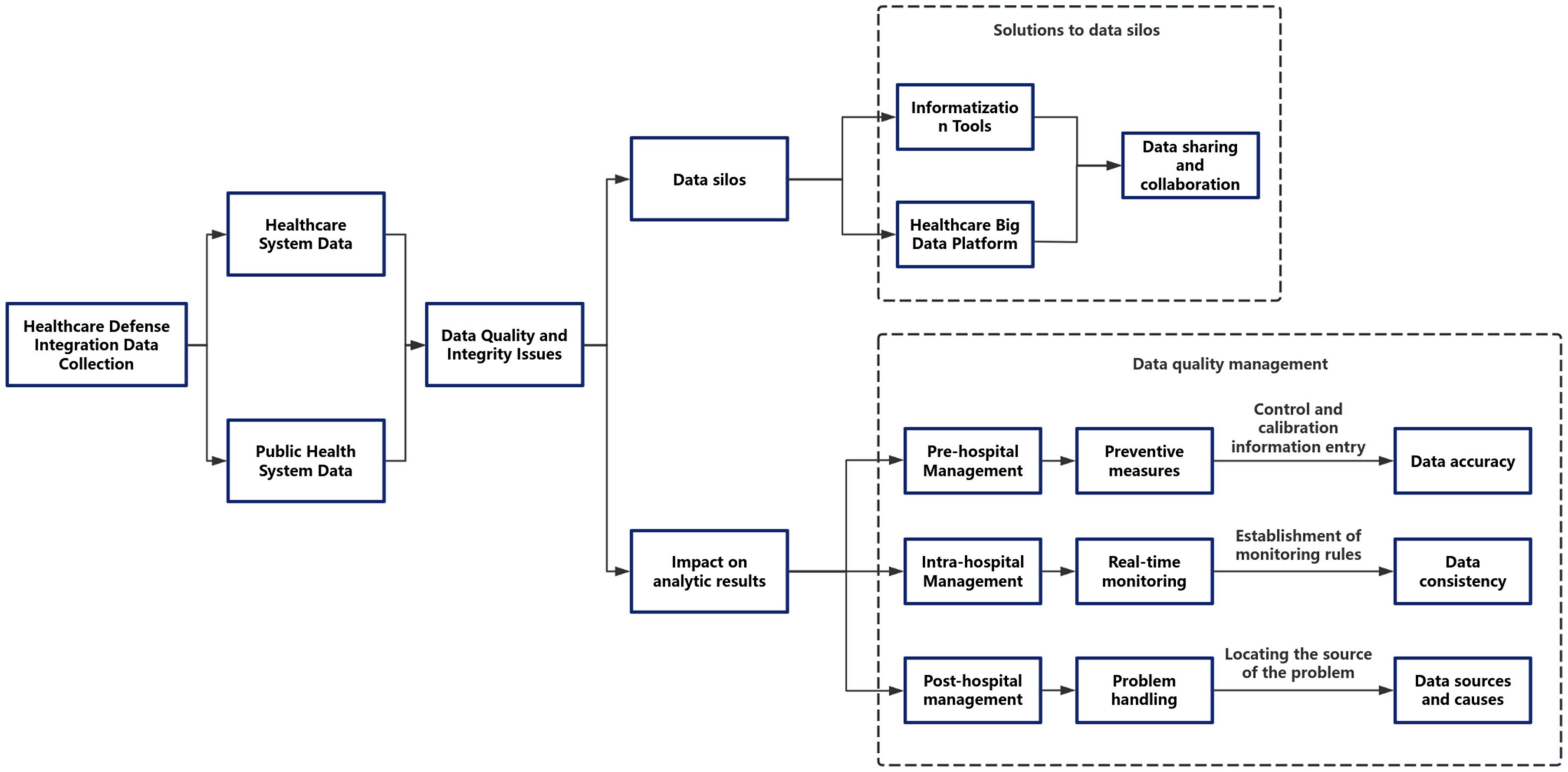
Healthcare prevention fusion data prototype diagram.

In data sovereignty zones, such as EU member states with strict data laws, edge computing nodes can be deployed to process data locally. Combined with homomorphic encryption technology for cross-regional analysis, this approach ensures data security while meeting the legal requirements of different regions. Finally, the development of AI-driven dynamic consent management portals can adjust data usage permissions according to regional laws, allowing patients to more flexibly control the use of their data. For example, Chinese patients can choose to participate in GDPR-compliant research projects through granular consent forms (see Figure 3).

### 1.4 Risk prediction models in chronic disease management (pre-hospital)

Chronic diseases, as complex conditions influenced by multiple factors, involve not only genetics, medical conditions, social conditions, and climate but are also closely related to individual lifestyle choices. According to the World Health Organization, 60% of the causes of chronic diseases are attributable to personal lifestyle (1), such as unhealthy diet, insufficient physical activity, tobacco use, and harmful use of alcohol. These behavioral factors largely determine the occurrence and progression of chronic diseases. Big data can gather chronic disease health risk-related data from these sources, achieving multi-dimensional and comprehensive data collection. Risk prediction models for chronic disease health based on big data typically analyze population health/disease spectra and extract risk factors closely related to health/disease. These models can obtain average health risk curves for different age and gender groups, compare an individual’s absolute risk with the average risk of the corresponding group, determine personal disease risk levels, and identify risk factors leading to increased risk. This enables the development of scientifically sound personalized intervention guidelines, achieving primary and secondary prevention in chronic disease management (13).

AI can identify new hypertension genes through machine learning algorithms and evaluate patients by integrating various parameters such as stages of hypertension, blood pressure control, and accompanying comorbidities, thereby achieving early diagnosis and prevention. For example, Ye et al. (14) used personal patient electronic health record (EHR) datasets from a statewide health information exchange network and employed the XGBoost machine learning algorithm for feature selection and model construction. The risk model for developing hypertension within one year achieved an AUC (area under the curve) of 0.917 and 0.870 in retrospective and prospective cohorts, respectively. The prospective validation demonstrated an accurate one-year risk prediction model for predicting primary hypertension, providing insights for hypertension and related disease interventions, and improving hypertension care.

Subsequently, KANEGAE Hiroshi (15) used health check-up data from 18,258 individuals from 2005 to 2016 to develop a highly accurate prediction model for future hypertension in the general population using machine learning (ML) algorithms. ML-based analysis allowed all BP measurements to be incorporated into the same model, resulting in superior performance in predicting new-onset hypertension compared to XGBoost and logistic regression models. They developed a highly accurate future hypertension prediction model in a generally normotensive population using machine learning methods, which can identify high-risk individuals and promote early non-pharmacological interventions. Similarly, Yan-Hui Li and colleagues analyzed gene expression in hypertensive patients, identifying 177 new hypertension genes through machine learning algorithm development. This represents a novel approach to achieving secondary prevention of chronic diseases.

In other chronic disease contexts, Kupersmith et al. (16) explored the impact of additional attributes using electronic health record data to identify a high comorbidity rate of mental disorders (24.5%) among diabetes patients, aiming to conduct risk prediction studies for diabetic patients. Similarly, McCoy et al. (17), conducted a retrospective analysis using data from the Optum laboratory data warehouse, finding that intensified treatment nearly doubled the risk of severe hypoglycemia in complex patient cases. Jelinek et al. (18) applied data mining algorithms to large clinical datasets and discovered that including oxidative stress biomarkers increased the classification accuracy of type 2 diabetes mellitus (T2DM) from 78.71 to 86.64% at an HbA1c level of 6.5%. Including interleukin-6 in the algorithm, but at a lower optimal HbA1c range of 5.73 to 6.22%, improved T2DM classification accuracy to 85.63%, significantly enhancing the risk prediction capability for diabetes patients. In 2019, based on clinical data from the Yinzhou District Health Information System, Wang et al. (19) from Peking University developed a predialysis chronic kidney disease risk assessment model. Serbanati (20), using neural networks and logistic regression analysis techniques, established predictive models for chronic disease patients, providing effective prediction for conditions such as hypertension and diabetes.

### 1.5 Clinical prevention in chronic disease management (in-hospital)

With the advancement of technology, an increasing number of chronic disease management applications and related systems have emerged. This has led to the diversification and variety of patient health data sources. However, due to the heterogeneity of the data, the more data there is, the more difficult it becomes to analyze. By continuously improving methods in data mining, the efficiency of chronic disease management and the accuracy of diagnosis and treatment have significantly improved. Shunda et al. (21). Proposed a deep learning-based medical auxiliary diagnosis data processing method to address the lack of efficient and accurate analysis methods for massive medical diagnosis data. They utilized neural networks deploying multilayer perceptrons to analyze preprocessed data, thereby achieving diagnostic classification and providing intelligent auxiliary diagnosis for doctors. The proposed method’s loss value and average accuracy rate were 53 and 85%, respectively, both outperforming other comparative methods. Hu et al. (22) proposed a novel approach that combines network analysis and machine learning to predict the length of stay (LOS) for older adult patients with chronic diseases. They constructed two networks: a multimorbidity network (MN) and a patient similarity network (PSN), and developed innovative network features. Five machine learning models with different input feature sets (extreme gradient boosting, gradient boosting decision tree, random forest, linear support vector machine, and deep neural network) were developed to compare their performance. The inclusion of network features significantly improved the performance of the prediction models, demonstrating the practicality of MN and PSN for LOS prediction. This underscores the potential value of network-based machine learning in chronic disease management.

In the medical data analysis of chronic diseases, many researchers have applied association rule data mining techniques and achieved significant experimental results. Xiaobing et al. (23). Utilized the numerical attribute characteristics of aggregation algorithms to discretize datasets and divide them into several optimized datasets, mining useful association rules from tumor diagnosis data. This provides important reference value for the clinical diagnosis of chronic tumors. Ningning et al. (24). Used classification algorithms to analyze data from patients with type I diabetes, discovering that the generated classification association rules were highly consistent with medical research findings. Classification association rule technology has a solid theoretical foundation in chronic diabetes research. Among these, the Apriori algorithm in association rules is the primary algorithm used in data mining for chronic disease treatment and prevention. Zheng (25). summarized the traditional Apriori algorithm, finding that its biggest drawback is the need to repeatedly scan the database to obtain frequent itemsets, which inevitably affects the efficiency of data mining and consumes a large amount of memory. In contrast, Liu et al. (26) proposed an association rule data mining algorithm that combines clustering matrix and pruning strategies. This algorithm compresses the stored transaction database using the clustering matrix method and introduces pre-pruning and post-pruning strategies based on adding constraint conditions, improving the algorithm. Its execution time is significantly lower than that of the traditional Apriori algorithm, enhancing the efficiency of data analysis for chronic disease treatment and prevention.

### 1.6 Accurate chronic disease management (post-hospital)

By comprehensively utilizing risk prediction models and other tools for chronic disease management, a new integrated medical prevention service model that optimizes the pre-hospital, in-hospital, and post-hospital stages, and incorporates the four levels of prevention, can be achieved for precise chronic disease management. The refined management of chronic diseases aims to improve the management rate, control rate, and hospitalization rate of patients with chronic and special diseases, ultimately enhancing their quality of life. For example, the Shanghai Center for Disease Control and Prevention has explored establishing an integrated and precise chronic disease health management service model (27), achieving integrated whole-process health management centered around individuals in the pre-hospital stage. Using big data capture and matching, the system reminds doctors to provide screening services and automatically tracks clinical diagnosis information, forming a closed-loop management system for chronic disease screening services.

In-hospital, McManus et al. (28) conducted the HOME BP study, a randomized controlled trial of home online blood pressure management and evaluation. This study randomly assigned 622 patients with poorly controlled blood pressure to either an internet medical platform intervention group or a conventional treatment group. After a median follow-up of one year, the internet medical intervention group showed a reduction in systolic blood pressure of 3.4 mmHg compared to the conventional treatment group. Yangyang and Xingdong (29) proposed a new clustering method combining agglomerative hierarchical clustering and Gaussian mixture models to effectively handle dynamic missing data, which can identify cases of masked hypertension.

Post-hospital, early screening of high-risk atrial fibrillation (AF) populations based on internet medical platforms also improves early detection rates and timely intervention for AF. The Huawei Heart Study (30), which uses photoplethysmography (PPG) technology for high-risk AF screening, recruited 187,912 patients and monitored their heart activity remotely. The positive predictive value for AF was 91.6%. Another study by Perez et al. (31) collected electrocardiogram data from 419,297 participants uploaded through Apple smartwatches, resulting in a positive predictive rate for AF of 84%, providing significant data support for post-hospital follow-up.

### 1.7 Health management within the integration of medicine and defense

Health management in the integration of medicine and defense covers the entire lifecycle of individuals, from health promotion and disease prevention to treatment, rehabilitation, and long-term care. It provides timely and effective treatment and rehabilitation services for those already afflicted, and offers long-term monitoring and management for chronic disease patients to improve their quality of life. The concept of “integrating medicine and defense” combines disease treatment and prevention (3), integrating medical and preventive services to effectively link and synergize them, thereby minimizing the occurrence of health problems, targeting the control of health deterioration, enhancing the appropriateness and effectiveness of healthcare services, and achieving the goal of “putting health at the center” (32).

After the COVID-19 pandemic, communities and public hospitals have gradually shifted from a “disease-centered” approach to a “people-centered health” approach, no longer prioritizing medical treatment but also emphasizing prevention, especially for chronic diseases, to achieve early detection, diagnosis, and treatment. The aim is to construct a more efficient and coordinated healthcare system, reducing disease burden through prevention and improving treatment outcomes through medical care. Driven by the Healthy China strategy, regions have actively explored the integrated development model of medicine and defense: Jia et al. (33) used methods such as literature analysis, policy summarization, and expert interviews to preliminarily establish an evaluation index system for on-site assessment of integrated medical and preventive services for chronic diseases in grassroots healthcare institutions, enhancing the capabilities of urban and rural community health service institutions in integrated medicine and defense, providing them with references and bases. In Wuhan, community service stations have focused on enhancing grassroots service capabilities (34), continually refining work mechanisms, promoting family doctor contract services, and comprehensively enhancing integrated medicine and defense capabilities at the grassroots level. In Sanming City (35), under government leadership, basic medical insurance funds have been merged with basic public health expenses, encouraging healthcare institutions to provide integrated medicine and defense services, and through network construction, system development, and institutional improvement in parallel, they have preliminarily established a basic framework for integrated medicine and defense, transitioning from separated medicine and defense to integrated reforms. Yang Jiang city established the first national-level public health hospital at the prefecture-level, integrating multiple institutions and departments, responsible for the medical, preventive, and rehabilitation management of infectious, mental, chronic diseases, and other businesses across the city. The hospital has promoted the integration of prevention and clinical medicine, improving the collaborative efficiency of prevention and treatment through the participation of medical personnel in disease monitoring. In addition, some city hospitals (such as West China Hospital of Sichuan University) have collaborated with grassroots healthcare institutions to establish integrated medicine and defense centered on health management.

**Figure 4:**
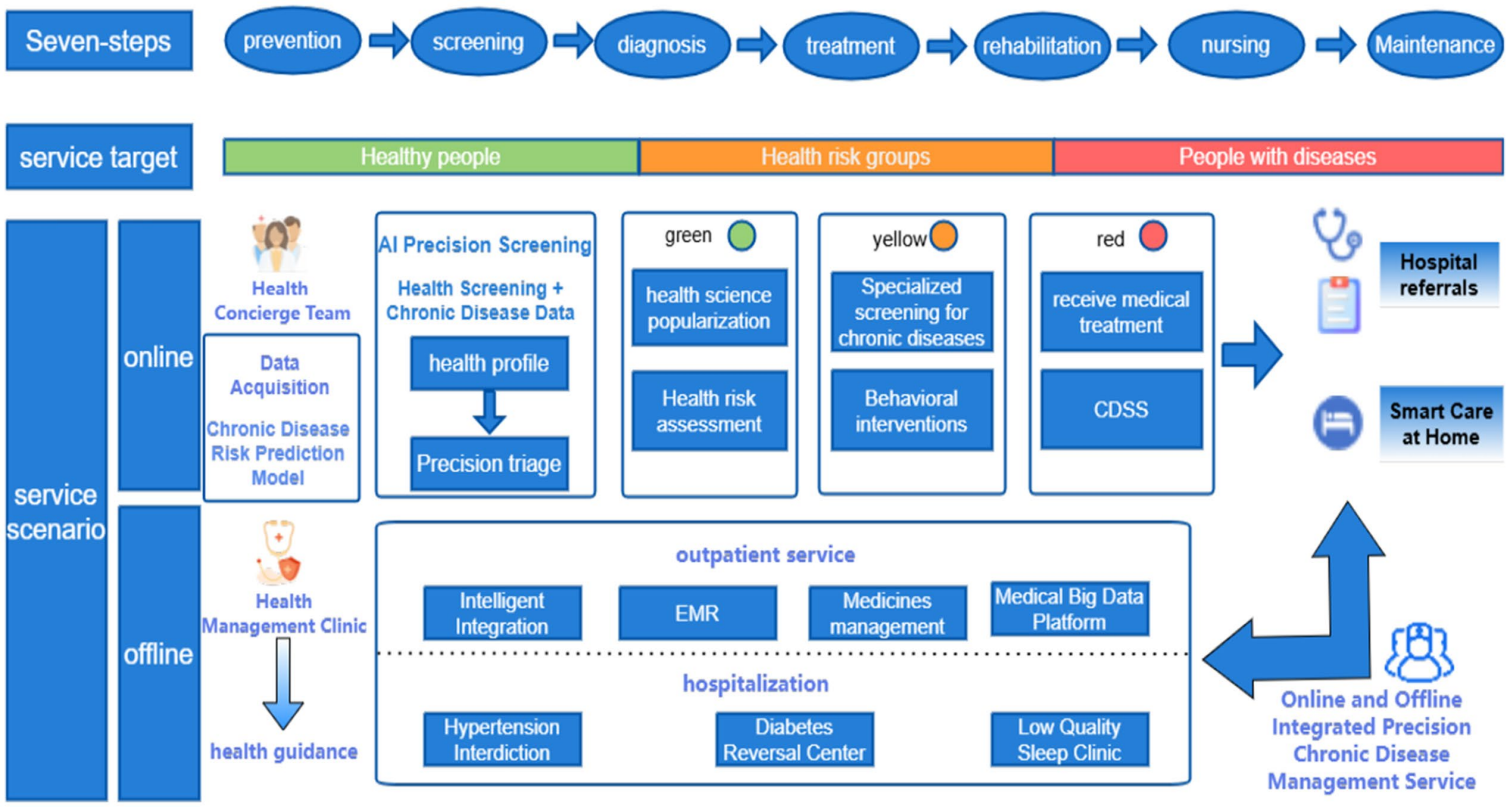
Prototype architecture diagram for precision health management integration within a public hospital system.

Figure 4 illustrates the prototype architecture of precision health management integration within China’s public hospital system, demonstrating a novel “online-offline convergence” care delivery model. This paradigm establishes a seven-phase integrated framework (prevention, screening, diagnosis, treatment, rehabilitation, nursing, and maintenance) that orchestrates closed-loop health management (36) through coordinated institutional referrals and AI-powered home care platforms. The system leverages interoperable health information exchanges to synchronize multisource medical data across the entire care continuum—from primordial prevention to post-rehabilitation monitoring. Key operational objectives include: (1) Enhanced population health metrics through predictive risk stratification algorithms; (2) Improved early detection rates for non-communicable diseases via multimodal screening integration (≥40% sensitivity gain versus conventional methods); (3) Reduced critical illness incidence through precision lifestyle interventions (targeting 15–20% risk reduction in cardiometabolic disorders). Implementation of blockchain-secured care pathways and federated learning architectures ensures real-time care coordination while maintaining GDPR-compliant data governance. This patient-centric model optimizes resource allocation efficiency (demonstrating 30–35% reduction in redundant diagnostics) and enhances care continuum personalization through adaptive neural recommendation engines, representing a significant advancement in value-based healthcare delivery systems.

### 1.8 Regional disparities and multilevel differentiation in chronic disease management across China

While China’s chronic disease management system has achieved notable progress through policy initiatives and technological advancements, significant regional disparities and structural differentiation persist due to uneven economic development, resource allocation, policy implementation efficacy, and technological adoption. These variations manifest across service models, management efficiency, patient engagement, and health outcomes, as summarized in Table 1.

**Table 1:** Comparison of regional differences in chronic disease management in China.

Comparison dimension	Eastern developed_regions	Western underdeveloped_regions	Urban vs. rural comparison
Information technology level	High: Integrated health management platforms enable full-cycle closed-loop management.	Low: Reliance on manual registration and follow-up, lack of data collection equipment.	Urban: Data platforms are widespread, remote monitoring technology is mature; Rural: Information silos, severe data fragmentation.
Policy implementation	Comprehensive: Integration of medical insurance and public health funding	Lagging: Grassroots focus on treatment over prevention, difficulties in policy implementation.	Urban: High coverage of family doctor contract services; Rural: Insufficient fiscal investment, contract services lack sustainability.
Technology Application	Advanced: AI-assisted diagnosis, machine learning risk prediction models	Backward: Reliance on manual records, limited application of risk models.	Urban: Smart wearable devices are popular (e.g., PPG technology for atrial fibrillation screening); Rural: Lack of technical training and equipment support.
Grassroots Service Capacity	Strong: Community health centers provide personalized interventions (e.g., Shanghai model).	Weak: Focus on simple follow-ups, lack of data analysis capabilities.	Urban: Multidisciplinary collaboration for precise management (e.g., West China Hospital); Rural: Chronic disease management remains superficial.
Patient Participation	High: Mobile health applications are widespread, active participation in prevention	Low: Low health literacy, late-stage medical visits.	Urban: Health education coverage >80%; Rural: Limited access to information, low awareness of prevention.
Economic and Insurance Support	Strong: High medical insurance reimbursement rates, support for long-term management (e.g., Sanming integrated insurance model).	Weak: High out-of-pocket costs, heavy burden on low-income groups.	Urban: Pilot programs for differentiated payment mechanisms; Rural: Inadequate insurance coverage, unsustainable health management.

#### 1.8.1 Geographical imbalance in development

Coastal regions, exemplified by Shanghai, lead in digital health integration and clinical-public health convergence. Shanghai’s unified health management platform enables closed-loop chronic disease management spanning screening, diagnosis, and follow-up (27). In contrast, western rural areas face systemic challenges, including insufficient medical resources and outdated infrastructure. Township hospitals in provinces like Gansu often lack basic chronic disease monitoring equipment, hindering risk prediction model deployment (37). Compounding this issue, central-western regions (e.g., Sichuan) with aging populations exhibit higher chronic disease burdens but receive inadequate training and technical support for primary care providers.

#### 1.8.2 Heterogeneity in policy implementation

Decentralized execution of national policies creates divergent local practices. Sanming City’s integrated “prevention-treatment-management” framework, funded through pooled medical insurance and public health budgets, significantly improved hypertension and diabetes control rates (35). Conversely, provinces prioritizing curative over preventive services demonstrate fragmented care coordination. Wuhan’s family physician contracting system enhanced grassroots prevention capabilities (34), while fiscally constrained regions struggle to sustain such programs due to insufficient funding.

#### 1.8.3 Hierarchical fragmentation in healthcare delivery

Tertiary hospitals dominate complex case management and predictive model development—West China Hospital’s multidisciplinary approach exemplifies advanced care for refractory chronic diseases. However, primary care institutions, intended as frontline prevention hubs, remain hampered by workforce shortages and data silos. Surveys reveal community health centers predominantly conduct rudimentary follow-ups rather than personalized interventions (33), exacerbating patient influx to overcrowded tertiary facilities and undermining sustainable prevention.

#### 1.8.4 Technological adoption gaps

AI-driven diagnostics and remote monitoring thrive in eastern metropolitan areas. Huawei’s photoplethysmography-based atrial fibrillation screening (30) demonstrates mature applications in first-tier cities. Meanwhile, central-western primary care facilities rely on manual records due to inadequate data infrastructure and training, perpetuating information fragmentation. Remote regions face additional barriers in predictive modeling due to sparse, non-interoperable health data (37).

#### 1.8.5 Socioeconomic determinants of patient engagement

Health literacy and financial capacity disparities critically influence outcomes. In the Yangtze River Delta, 62% of residents actively use mobile health apps for self-management, compared to <18% in western rural areas (38). Economic constraints drive delayed care-seeking in impoverished regions, with late-stage disease presentation correlating to 3.2-fold higher mortality in diabetes cohorts. Regional variations in insurance reimbursement rates (45–85%) further limit access to sustained management for vulnerable populations.

Policy coordination and cross-regional cooperation play a crucial role in addressing regional differences in chronic disease management, as well as in coping with the lack of medical resources and lagging technological application in under-resourced regions (39). Firstly, establishing an effective policy coordination mechanism is crucial. The central government should formulate a unified national strategy for the balanced development of medical resources, clarifying the responsibilities and goals of each region in the allocation of medical resources and the promotion of technology. For example, a special fund for the balanced development of medical resources could be set up, with financial transfer payments tilted towards resource—deficient areas. Local governments need to develop detailed implementation plans based on the central plan and local realities to ensure the effective use of funds and the smooth progress of projects. Meanwhile, a cross—departmental coordination body, such as a Medical Resources Coordination Committee involving health, finance, and science and technology departments, should be established to hold regular meetings, communicate information, and resolve issues in policy implementation.

Cross—regional cooperation is also an effective way to improve the medical level in resource—deficient areas (40). Developed regions can establish medical support cooperation relationships with resource—deficient areas. This cooperation can be carried out in a hospital—to—hospital manner, for instance, with top—tier hospitals in major cities forming partnerships with county—level hospitals in remote areas. Hospitals in developed regions can send medical teams to resource—deficient area hospitals on a regular basis for consultations, surgical guidance, academic lectures, and other activities, while also accepting medical staff from resource—deficient area hospitals for further study. In addition, remote medical cooperation can be carried out, using Internet technology to realize functions such as remote diagnosis and remote training, breaking geographical barriers and enabling patients in resource—deficient areas to access high—quality medical services.

In terms of technical assistance and training programs, the government should increase investment in resource—deficient areas. On the one hand, medical equipment aid projects can be launched, donating or providing advanced medical equipment at preferential prices to resource—deficient areas according to local medical needs, such as digital X—ray machines and ultrasound diagnostic equipment, along with equipment installation, debugging, and maintenance services. On the other hand, large—scale medical staff training programs should be implemented. Special training funds can be set up to conduct stratified and categorized training for medical personnel in resource—deficient areas. For example, for primary—level medical staff, general medicine training can be carried out to improve their ability to diagnose and treat common and frequent diseases; for specialist doctors, new technology and new therapy training can be provided, such as minimally invasive surgery training. The training methods can combine online and offline approaches, with online network course platforms providing a wealth of learning resources for medical staff to study anytime and anywhere, and offline centralized training and practical operation training to ensure training effectiveness (41).

Through these policy coordination mechanisms, cross—regional cooperation, and technical assistance and training programs, it is expected to gradually narrow the gap in medical resources and technology application capabilities between regions, alleviate social inequality, and improve the overall medical level in remote and resource—deficient areas, providing local residents with higher—quality and more equitable medical services.

## 2 Discussion

In recent years, the National Health Commission and the China CDC have actively promoted the integration of medical and preventive care, aiming to establish a disease prevention and control system with professional public health institutions as the backbone, medical institutions as the support, and primary healthcare institutions as the foundation. Various regions have conducted effective explorations in this integration, achieving initial successes. However, a persistent “treatment-over-prevention” mindset remains prevalent among both healthcare providers and patients (38). Historically, the healthcare system has prioritized disease treatment over prevention, leading to relatively lower resource allocation and attention to public health services. This issue is not solely attributable to medical institutions; uneven health literacy and misconceptions about prevention among the public also contribute to a greater reliance on curative care. Overall, the health needs of most Chinese residents remain disease-oriented. Addressing this requires leveraging big data to analyze patients’ lifestyles, health statuses, and disease risks, providing personalized health education to help patients better understand and manage their health.

On the other hand, the lack of effective integration between medical information systems and public health information systems has resulted in data silos, leading to resource waste and increased management complexity. Disparate information platforms across medical institutions and primary care facilities hinder data sharing. Current public health surveillance systems, including infectious disease monitoring, chronic disease management, and health risk factor surveillance, operate independently in many regions without integration, preventing unified archiving of resident health information. The incompleteness and inconsistency of resident health data (42), coupled with the absence of unified chronic disease prevention management and integrated medical-preventive care pathways, often result in redundant follow-ups and health management for the same chronic disease patient across community health centers and large public hospitals. This not only leads to duplicated investments and resource waste but also compromises the continuity and consistency of care experienced by patients (43).

Chronic disease risk prediction models are one of the effective measures for controlling chronic diseases (44). However, current research on these models, both domestically and internationally, faces limitations in variable selection and algorithm application, restricting their predictive accuracy and ability to comprehensively capture the complex factors influencing chronic disease risks. To enhance the interpretability and practicality of these models, future research needs to focus on data integration, variable analysis, algorithm diversification, and model personalization.

Moving forward, research in China’s medical-preventive care integration should focus on the following areas to advance the scientific, precise, and intelligent development of chronic disease management:

(1) Data integration and sharing (45): Establishing unified data standards and exchange protocols to break down barriers between medical and public health information systems, enabling seamless multi-source data integration. Developing national or regional data-sharing platforms that consolidate clinical data, health monitoring data, environmental data, and other multidimensional information will provide comprehensive support for chronic disease prevention and control. Innovations in data security and privacy protection technologies will also be critical to ensure the safety and compliance of resident health information. In turn, data standards will evolve through the following research pathways

Phase 1 (foundation building): Conduct a comprehensive survey of the current medical data status in different regions. This includes data formats, data sources (such as hospitals, clinics, and medical laboratories), and data quality. Based on the survey results, establish a basic data standard framework. For example, unify the data formats for patient demographics (name, age, gender, etc.), medical history records, and basic examination results (such as blood pressure, blood glucose levels). Set up a data standardization working group composed of medical informatics experts, data engineers, and representatives from medical institutions to oversee the implementation.

Phase 2 (expansion and integration): Expand the data standardization to more complex medical data types. This includes medical imaging data (X-rays, CT scans, MRI images), electronic medical records (EMRs), and medical device data (such as data from pacemakers and implantable glucose monitors). Develop data integration standards to enable the seamless integration of data from different sources. For example, establish a standard for the integration of data from different hospital information systems (HIS) and laboratory information systems (LIS).

Phase 3 (optimization and sustaining): Continuously monitor and optimize the data standardization process. Set up a feedback mechanism to collect feedback from medical staff, data users, and patients. Use advanced data quality assessment tools to regularly check the quality of standardized data. Make adjustments to the data standards according to the latest medical research findings and technological advancements.

(2) Optimization of chronic disease risk prediction models: Future research should emphasize the integration of multi-source data, including clinical data, genomic data, lifestyle data, and social environmental data, to comprehensively capture the complex factors influencing chronic disease risks. Exploring diverse machine learning algorithms, such as deep learning, ensemble learning, and reinforcement learning, will enhance the predictive accuracy and generalizability of these models. Furthermore, the development of personalized risk prediction models tailored to individuals’ biological characteristics, behavioral habits, and environmental factors will become a key trend.

Step 1 (algorithm development and optimization): Focus on developing efficient federated learning algorithms that can handle the heterogeneity of medical data. Medical data from different regions may have different distributions due to differences in patient populations, medical practices, and data collection methods. Research on how to optimize the federated learning algorithms to deal with such data heterogeneity is crucial. For example, develop algorithms that can weight the data from different regions according to their data quality and representativeness.

Step 2 (security and privacy enhancement): Ensure the security and privacy of medical data in the federated learning process. Although federated learning does not share the raw data, there is still a risk of data leakage through the model parameters. Research on advanced encryption techniques, such as homomorphic encryption and secure multi—party computation, to protect the data during the model training and communication process.

Step 3 (performance evaluation and comparison): Establish a comprehensive performance evaluation system for federated learning models in the medical field. Compare the performance of federated learning models with traditional centralized learning models in terms of accuracy, robustness, and generalization ability. For example, in a cross—regional disease prediction task, evaluate how the federated learning model performs compared to a model trained on the combined data of all regions (if data sharing were possible).

(3) Health literacy improvement and innovative health education (46): Future research should explore new models of health education based on big data and AI, delivering precise health knowledge and personalized recommendations to improve public health literacy and self-management capabilities. Strengthening community participation and the family doctor system will also be crucial, building community-based health management networks to shift the focus of chronic disease prevention and control upstream.

Data collection and analysis: Collect a wide range of health—related data from different regions. This includes demographic data (such as age, gender, occupation), medical history, lifestyle habits (such as diet, exercise, smoking and drinking habits), and health—related behavioral data (such as the frequency of medical visits, the use of health—care products). For example, through online questionnaires, mobile health apps, and medical record systems, a large amount of data can be collected. Then, use big data analysis techniques to mine the potential health needs and problems of different population groups in different regions. For instance, in a certain area, it may be found through data analysis that the incidence of a certain chronic disease is high among the older adult, and their knowledge and self—management ability of this disease are relatively weak.

Artificial intelligence—based knowledge delivery and suggestion: Develop an artificial intelligence—driven health knowledge delivery and suggestion system (47). Based on the analysis results of the collected data, the system can precisely push health knowledge and personalized health suggestions to different users. For example, for a young office worker in an urban area who has a sedentary lifestyle and a high—fat diet, the system can push knowledge about the prevention of cardiovascular and cerebrovascular diseases, such as the importance of reasonable diet and regular exercise, and personalized suggestions such as a suitable exercise plan and a healthy diet recipe. At the same time, the system can also use artificial intelligence technology such as natural language processing to provide users with interactive health consultation services, answering their health questions in a timely and accurate manner.

Cultural assessment and content customization: Conduct a comprehensive cultural assessment in different regions. Understand the local cultural beliefs, values, and traditional health—related concepts. For example, in some rural areas, traditional Chinese medicine has a deep cultural foundation. In the health literacy improvement plan, more content about traditional Chinese medicine health preservation can be included, such as the use of traditional Chinese medicine diet and the principles of traditional Chinese medicine massage. In addition, the language and expression of health education materials should also be adapted to the local cultural habits. For example, in some ethnic minority areas, use the local ethnic language and vivid local dialects to explain health knowledge, so as to make it easier for residents to accept and understand.

Resource—based education method selection: According to the resource differences of different regions, select appropriate health education methods (48). In areas with abundant medical resources, such as large cities, more advanced health education methods can be used. For example, organize health lectures by well—known medical experts in hospitals and communities, and use virtual reality (VR) and augmented reality (AR) technologies to carry out immersive health education experiences, such as simulating the process of surgery or the spread of diseases in the human body. In resource—poor areas, such as remote mountainous areas, more simple and practical health education methods can be used. For example, train local health volunteers to carry out door—to—door health education and distribute simple and easy—to—understand health brochures.

**Figure 5:**
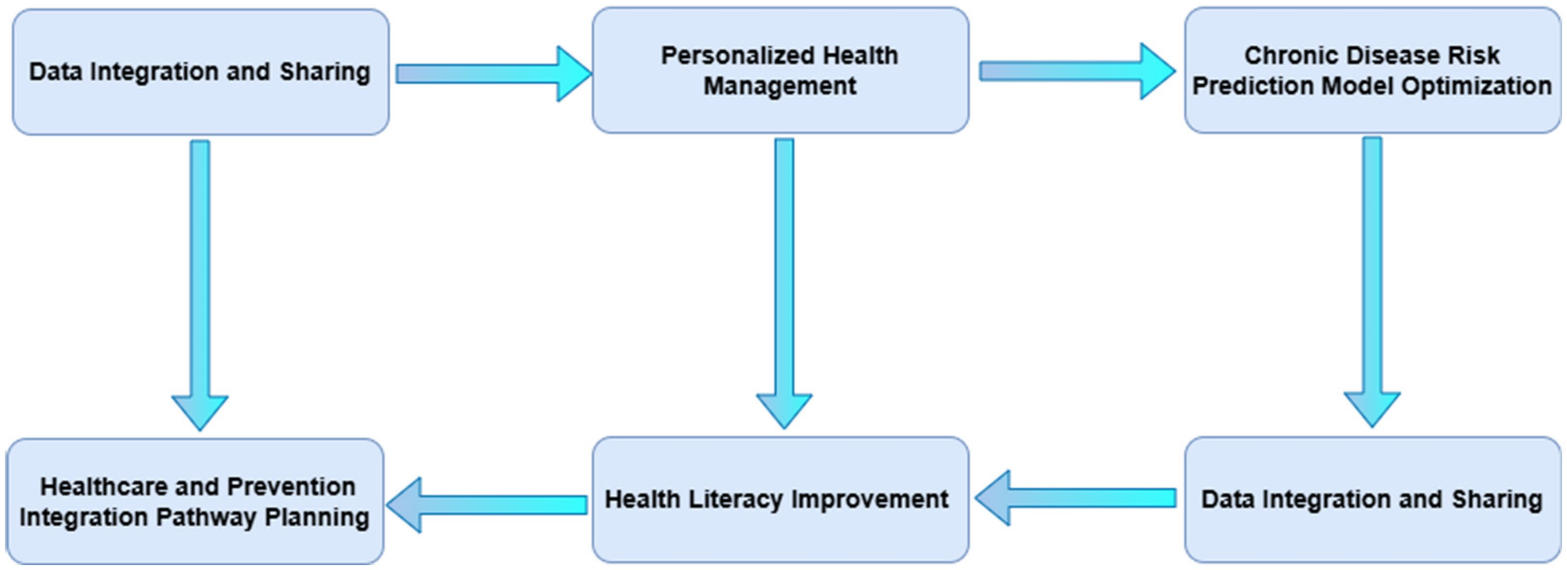
Roadmap for development in the field of healthcare prevention integration.

By addressing these challenges and focusing on these research priorities, China can advance the integration of medical and preventive care, ultimately improving the management and outcomes of chronic diseases (see Figure 5).

## 3 Conclusion

In the context of big data, significant progress has been made in the informatization of chronic disease health management. This study explores optimized pathways for chronic disease management from the perspective of medical-prevention integration, demonstrating that big data technologies will serve as the core driver for future integration efforts, particularly through the following strategies:

(1) Data-driven scientific decision support: utilizing multidimensional data analysis (e.g., clinical records, environmental factors, and regional epidemiological patterns) to provide evidence-based insights for chronic disease prevention, diagnosis, and management. For instance, comparative analysis of regional disease prevalence enables prioritized deployment of screening and intervention resources in high-risk areas.
(2) Predictive analytics and targeted interventions: integrating machine learning with spatiotemporal data analysis to develop dynamic risk prediction models. These models facilitate early identification of high-risk populations across regions and support the design of geographically tailored intervention pathways aligned with local healthcare resource availability.
(3) Mobile health and remote monitoring systems: deploying smart wearable devices and telemedicine platforms to continuously track patients’ physiological metrics and behavioral data, thereby establishing personalized health management “tracking pathways” to enhance treatment adherence and care continuity.
(4) Integrated regional health information platforms: establishing cross-institutional and cross-regional chronic disease management platforms to unify clinical records, health profiles, and public health data. Such platforms enable comparative analysis of regional management outcomes (e.g., disparities in diabetes control efficacy) and promote resource optimization through interregional knowledge sharing.
(5) Behavioral interventions and health communication: designing region- and population-specific health education programs based on patient data profiling. Information platforms monitor behavioral improvement trajectories (e.g., exercise habits, medication adherence) to dynamically refine intervention strategies.

By enhancing data-driven regional collaboration, process tracking, and precision decision-making, these strategies will substantially improve the efficacy of medical-prevention integration in chronic disease management, shifting the paradigm from “fragmented treatment” to “comprehensive prevention and control.” This transformation not only improves patients’ quality of life and reduces healthcare system burdens but also advances holistic optimization of chronic disease management through interregional data benchmarking and standardized best practices. Ultimately, it achieves multidimensional integration of services, data, and resources, offering a scalable framework for building resilient public health systems.
